# Effects of Production Method and Repeated Freeze Thaw Cycles on Cytokine Concentrations and Microbial Contamination in Equine Autologous Conditioned Serum

**DOI:** 10.3389/fvets.2021.759828

**Published:** 2021-11-25

**Authors:** Josephine Hale, Kristopher Hughes, Sarah Hall, Raphael Labens

**Affiliations:** ^1^School of Agricultural, Environmental and Veterinary Sciences, Charles Sturt University, Wagga Wagga, NSW, Australia; ^2^Animal & Veterinary Sciences Department, Scotland's Rural College, Edinburgh, United Kingdom

**Keywords:** ACS, osteoarthritis, IRAP, horse, thermal stability

## Abstract

Autologous conditioned serum (ACS) is a common intra-articular treatment for osteoarthritis in horses. The objective of this study was to investigate the influence of ACS preparation method on product contamination and concentrations of relevant cytokines and the influence of multiple freeze/thaw cycles. Blood was obtained from 10 healthy Thoroughbred horses and processed in parallel using a commercial and a non-commercial method to obtain ACS. Fluorescent microsphere immunoassay (FMIA) analysis was performed to quantify Interleukin 1 receptor antagonist (IL-1Ra), Interleukin-10 (IL-10), Interleukin-1β (IL-1β) and tumor necrosis factor-α (TNF-α) concentrations in ACS obtained by both production methods. Effect of 3, 4 and 5 freeze/thaw cycles on concentrations of IL-1Ra, IL-10, IL-1β and TNF-α were assessed against baseline samples (2 cycles) in commercial ACS products. Standard aerobic and anaerobic culture methods were applied to both ACS products. Mixed effect one-way analyses of variance (ANOVA) were used to compare the two ACS production method for each cytokine. Repeated measures, mixed effect ANOVA were used to assess the effect of freeze/thaw on cytokine concentrations. Significance was set at *P* < 0.05. There was no difference in cytokine concentration between production methods (IL-1Ra *P* = 0.067, IL-1β *P* = 0.752, IL-10 *P* = 0.211 and TNF-α *P* = 0.25). Microbial growth was only observed in two samples obtained using the commercial production method. When compared to baseline, IL-1Ra concentration was decreased following the 5th freeze/thaw cycle (*P* < 0.001). These results suggest that the concentration of important cytokines are not influenced by ACS production method. When storing ACS samples for future use, freeze/thaw cycles associated with standard clinical practice are unlikely to influence cytokine concentrations. However, the lack of outcome measures associated with 1 or 2 freeze/thaw cycles represents a limitation of this study.

## Introduction

Osteoarthritis contributes to ~60% of all lameness conditions in the horse (1). This condition has considerable economic impact on racing and performance horse industries (2).While commonly used for the treatment of osteoarthritis, intra-articular corticosteroid administration is associated with an increased risk of musculoskeletal injury (3). Intra-articular administration of methylprednisolone acetate has also been shown to have deleterious effects on articular cartilage with cumulative use (4). Autologous conditioned serum (ACS) is an alternate, biological treatment for osteoarthritis that relies on specific conditioning of blood to generate an injectable product that contains increased concentrations of naturally occurring anti-inflammatory cytokines. Tumor necrosis factor α (TNF-α) and Interleukin 1β (IL-1β) are potent activators of intra-articular inflammatory pathways and synovial concentrations correlate with joint disease in humans and horses (5–7). Consequently, inhibiting the action of IL-1β represents a valuable therapeutic target in clinical patients (8). As such, *in vivo* administration of Interleukin 1 receptor antagonist (IL-1Ra), exerts a dose dependent anti-inflammatory and chondroprotective effect attributable to competitive receptor inhibition in humans and horses (9–12). When compared to triamcinolone, ACS has been shown to result in a 10-fold greater downregulation of IL-1β and TNF-α gene expression in co-culture of equine joint tissue (13). A commercial preparation kit for ACS is widely available to equine practitioners for intra-articular treatment of osteoarthritis and post arthroscopic interventions (11).

The production of ACS involves the incubation of venous whole blood with chromium sulfate (CrSO_4_) etched medical-grade glass beads at 37°C for 24 h, followed by the isolation of the serum. After filtration sterilization, a minimum of three ACS aliquots are typically frozen and stored, allowing repeat administration at later time points (8). However, the use of CrSO_4_ glass beads may not be critical and using uncoated blood collection tubes could suffice to produce therapeutic concentrations of IL-1Ra (14). Consequently, a simpler non-commercial method would be associated with cost benefits and the potential for an even greater use of biological products in equine veterinary practice. To justify clinical implementation of an alternate production method and to ensure safe use, an understanding of the effects of sample processing and storage is required. To avoid iatrogenic joint infections with ACS use, maintaining sterility throughout the production process is critical, particularly when utilizing open, non-commercial production systems, which may be more likely to result in inadvertent product contamination, in contrast to closed commercial systems.

As the beneficial therapeutic effects of ACS appear to be enhanced by repeated intra-articular administration, availability of ACS is essential (10, 15). Due to the complexity and time-consuming nature of the ACS production process, repeat collection for each treatment of an individual horse is typically not considered and current recommendations include the storage of ACS aliquots (3–5 ml) for future use. However, unintended defrosting of samples due to freezer breakdown, handling errors or shipment delays could jeopardize the effectiveness of ACS. Determination of the impact of freeze/thaw cycles on cytokine concentrations in equine ACS is necessary and to the authors' knowledge has not been described previously.

The first objective of this study was to compare the cytokine profiles of a commercial, “closed,” ACS production system and a non-commercial, “open,” ACS production method. We hypothesized that the products of both systems would have comparable profiles. The second objective was to compare microbial contamination of both production systems. We hypothesized that the open method would be associated with more frequent microbial contamination. The third objective was to investigate the effect of freeze/thaw cycles on concentrations of anti and pro-inflammatory cytokines. We hypothesized that increasing freeze/thaw cycles would result in a reduction in IL-1Ra, IL-10, IL-1β and TNF-α concentrations.

## Materials and Methods

### Animals

The study was approved by the Charles Sturt University Animal Care and Ethics Committee (Approval number A19023). Ten healthy Thoroughbred geldings, with a mean [± standard deviation (SD)] age of 9.5 (± SD 2.5) years (range 5–12 years) were included in this study.

### Sample Collection

For each horse, a total of 104 ml of blood was collected under aseptic conditions. Venipuncture sites were clipped, a 5-min scrub performed using sterile swabs in chlorhexidine solution (0.05%) before methanol-soaked sterile swabs were used to finalize preparation of the site. Using sterile gloves and a 21-gauge butterfly catheter, blood was collected via a single venipuncture of the left jugular vein. Blood was collected over a 1–2-min period, with minimal traction applied to the syringe plunger. The first 50 ml of blood was collected into a proprietary syringe (Orthokine IRAP, Dechra, Dusseldorf, Germany) containing the CrSO_4_ etched medical grade glass beads. An additional 50 ml of blood was collected into a 50 ml polypropylene syringe, from which the sample was transferred into a 50 ml polypropylene falcon tube (Sarstedt®) without the use of a laminar flow hood, for the non-commercial method of ACS production. A further 2 ml of blood, was deposited into an EDTA (BD Vacutainer®) and plain tube (BD Vacutainer®), for hematology and inflammatory blood profile analysis (for the concentrations of fibrinogen, serum amyloid A, total protein, albumin, globulin, and A:G ratio), respectively.

### Sample Preparation

**Figure 1:**
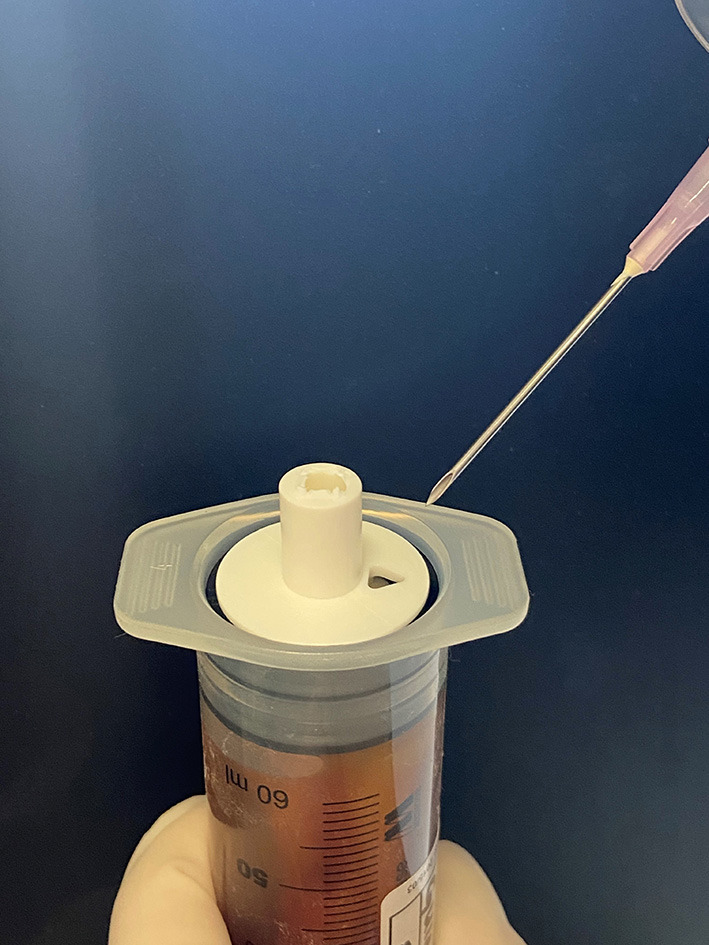
Triangular extraction port of the commercial autologous conditioned serum method.

Samples for both methods were incubated (“Melag 80,” Melag, Berlin, Germany) for 24 h at 37°C followed by centrifugation at 3,500 rpm for 10 min [Hermle Z300 centrifuge (Hermle Labortechnick GmBH, Whingen, Germany) for the commercial samples; Hettich Zentrifugen Rotofix 32 A, (Hettich Franke GmbH & Co, Balingen, Germany) for the non-commercial samples]. For the commercial method, the guide plate of the syringes was subsequently removed, and a 100 mm 18-gauge needle was inserted, through the triangular extraction port of the black stopper (Figure 1). The serum fraction was then slowly aspirated into a 30 ml polypropylene syringe as per the manufacturer's instructions. For each non-commercial sample, the screw cap was carefully removed from the Falcon tube and serum was aspirated using a 100 mm 18-gauge needle and 30 ml polypropylene syringe. For each sample, ~10 ml of serum was retrieved. Samples were subsequently sterilized using a single use filter with a 33 mm diameter and 0.22 μm pore size (Merck Millipore Ltd, Cork, Ireland) and divided into two sterile 5 ml polypropylene vials. One 5 ml aliquot was subsequently frozen at −80°C while the other aliquot underwent microbial culture. All frozen serum samples underwent an initial slow thaw process at 2°C after which the samples were re-aliquoted into 2 ml volumes (Sarstedt® Eppendorf tubes) utilizing sterile single use pipettes. The samples were then re-frozen and stored at −80°C until shipped on dry ice for molecular quantification. Total storage time for frozen samples did not exceed 18 months and shipped samples arrived at their destination frozen.

### Bacteriology

Standard methods for aerobic and anaerobic bacterial culture of ACS products were used and agar plates were read by sight by an experienced microbiologist to assess for bacterial colonies. Bacterial colonies recovered were identified by colony morphology, Gram stain (Australian Biostains) morphology, catalase (3% hydrogen peroxide) and oxidase (Oxidase Strips MB0266A) testing.

### Serum Analysis

A fluorescent microsphere immunoassay (FMIA) was performed on all serum samples. Cytokine measurements were optimized as a fourplex for IL-1Ra, IL-1β, IL-10 and TNF-α. The optimization of the multiplex plates was performed using the validation method described by Hall et al. (16), including polyclonal capture and detection antibodies (R&D systems' Minneapolis, Minnesota, United States) and magnetic carboxylated microspheres (BioRad, Hercules, California, United States). Information regarding the capture and detection antibodies is outlined in Table 1. A pooled serum sample was run undiluted and at dilutions of 1:2 and 1:4 for added control of linearity and recovery. The results for IL-1Ra at a dilution of 1:2 were greater than the detection range; however, all dilutions for IL-1β, IL-10, TNF-α were within the standard curve range. To allow all cytokines to be measured in undiluted samples, the range of standards used for IL-1β, IL-10, TNF-α was set at 41.0–10,000 and 81.9–20,000 pg/ml for IL-1Ra.

**Table 1:** Information on capture and detection antibodies used in this study including source and concentration.

**Cytokine**	**Bead region (BioRad catalog number)**	**Capture antibody (μg/ml)**	**Detection antibody (μg/ml)**	**Source**
IL-1ra	65 (MCA-0065-01)	Goat anti-equine 20	Biotinylated goat anti-equine 0.5	R&D Systems DY2466
IL-1β	77 (MCA-0077-01)	Goat anti-equine 20	Biotinylated goat anti-equine 0.5	R&D Systems DY3340
IL-10	35 (MC1-0035-01)	Goat anti-equine 20	Biotinylated goat anti-equine 0.5	R&D Systems DY1605
TNF-α	55 (MC1-0055-01)	Goat anti-equine 20	Biotinylated goat anti-equine 0.5	R&D Systems DY1814

For the freeze/thaw component of the study, sequential, repeated freeze/thaw cycles were performed on the commercial samples only. All samples were stored at −80°C until required. Samples were removed and placed in a rack at room temperature (16–18°C) until thawed. Samples remained at room temperature for 3 h during which time the analysis was performed. Samples were vortexed for 5–10 s before loading the samples into the FMIA assay plate and once again before refreezing. The minimum duration of freezing between thaws was 48 h. After each thaw, the samples were reanalysed by FMIA prior to refreezing at −80°C. In this investigation, the sample undergoing the second freeze/thaw represented the earliest observation and was considered the control in comparisons involving further freeze/thaw cycles.

### Statistical Analysis: Cytokine Concentrations in Commercial and Non-commercial ACS Preparations

To compare cytokine concentrations in the commercial and non-commercial preparations: mixed effect one-way analyses of variance (ANOVA) were performed using separate models fitted to each of the dependent variables; IL-1Ra, IL-10, IL-1β and TNF-α. Independent model effects were “Horse” (random effect, Horse 1–10) and “Method” (commercial vs. non-commercial).

### Statistical Analysis: Effect of Freeze/Thaw Cycles on Cytokine Concentrations

Repeated measures, mixed effect ANOVAs were performed with separate models fitted to each of the dependent variables; IL-1Ra, IL-10, IL-1β and TNF-α. The number of “Freeze/thaw cycles” was the repeated variable and “Horse” the random effect.

Prior to tests being performed, normality of raw data was assessed according to Shapiro-Wilk, same as for residuals post analyses. Residuals were plotted against predicted values to evaluate model fit and heteroscedasticity. If the data were nonparametric, inverse log and square root transformations were performed to meet test assumptions. Non-transformable data were analyzed using Friedman and Wilcoxon signed rank tests. *Post-hoc*, multiple comparisons of data were adjusted using the Bonferroni method (*P* < 0.008). Pearson and Spearman correlations were performed to assess the relationship between IL-1β and IL-1Ra and the mean or median IL-1β/IL-1Ra ratio was calculated for each container type. Tests were performed using SAS® on Demand for Academics (Cary, NC) with significance set at *P* ≤ 0.05. Depending on data distribution results were summarized using the mean ± standard deviation (SD) or median (interquartile range; IQR).

## Results

### Serum Analysis: Cytokine Concentrations in Commercial and Non-commercial ACS Preparations

The data for IL-1Ra, IL-1β and IL-10 required inverse log transformation to achieve normal distribution prior to analysis by ANOVA. Method of ACS production was not associated with a difference in IL-1Ra; IL-1 or IL-10 concentrations (*P* = 0.067, *P* = 0.752, *P* = 0.211, respectively). Analysis of non-parametric and non-transformable TNF-α data suggested no effect of production method (*P* = 0.25, Wilcoxon signed rank test). Descriptive statistics (median and IQR values) for each product type and molecule are provided in Table 2. Cytokine analysis inter-assay coefficient of variance for IL-1Ra, IL-1β, IL-10 and TNF-α were 5.7, 15.3, 5.9 and 11.2% respectively. The low and high cytokine analysis intra-assay coefficient of variance for IL-1Ra was 7 and 15.3%, for IL1-β was 8.0 and 7.7%, for IL-10 was 11.4 and 5%, and for TNF-α was 6.8 and 12.7% respectively. Validation of cytokine ranges is outlined in Table 3; Supplementary Materials 1, 2.

**Table 2:** Median (interquartile) concentrations for cytokines measured for each ACS production method.

**Container**	**Interleukin 1 receptor antagonist (pg/ml)**	**Interleukin 1β (pg/ml)**	**Interleukin 10 (pg/ml)**	**Tumor necrosis factor α (pg/ml)**
Commercial	3,616 (1,298–9,704)	1,657 (598–4,224)	60.89 (23.64–108.7)	6.27 (4.48–7.765)
Non-commercial	1,497 (500.3–3,375)	1,154 (641–2,242)	56.06 (29.36–124.9)	5.62 (4.48–7.773)

**Table 3:** Validation method for fluorescent microsphere immunoassay.

**Cytokine**	**Enzyme-linked immunosorbent assay duoset antibody ranges (pg/ml)**	**Cytokine ranges (pg/ml)**	**Validated**
IL-1Ra	300–20,000	81.9–20,000	**Supplementary Material 1**
IL-1β	125–8,000	41–10,000	**Supplementary Material 2**
IL-10	300–20,000	41–10,000	Hall et al. (16)
TNFα	31.2–2,000	41–10,000	Hall et al. (16)

For commercial and non-commercial containers, the median IL-1β/IL-1Ra ratio was 1:2.65 (IQR 1.72–3.13) and 1:1.34 (IQR 0.422–4.06), respectively. The commercial container data required a log transformation to achieve normal distribution, whilst the non-commercial container data was non transformable. A positive correlation between IL-1β and IL-1Ra was observed for commercial [*P*_Pearson_ = 0.008; *r* = 0.776 (95% CI 0.286–0.944), *R* squared = 0.602] but not for non-commercial containers (*P*_Spearman_ = 0.492). There was no difference in IL-1β/IL-1Ra ratio between the commercial and non-commercial containers (*P* = 1.0; Wilcoxon signed rank test).

### Bacteriology

Bacterial culture results for the non-commercial samples yielded no growth. Two of the commercial products yielded single colony growths of *Micrococcus* species and a non-haemolytic *Streptococcus* species, respectively.

### Serum Analysis: Effect of Freeze/Thaw Cycles on Cytokine Concentrations

The IL-1Ra data were non-normally distributed and required square root transformation. There was an association between the number of freeze/thaw cycles and IL-1Ra concentration (*P* < 0.001). Differences of least squares means revealed that as the number of freeze/thaw cycles increased, the concentration of IL-1Ra protein decreased (2 vs. 3 freeze/thaw cycles *P* = 0.253, 2 vs. 4 freeze/thaw cycles *P* = 0.065 and 2 vs. 5 freeze/thaw cycles *P* < 0.001).

The IL-1β data were non-normally distributed and required square root transformation. There was an association between the number of freeze/thaw cycles and the concentration of IL-1β (*P* = 0.046) but in adjusted *post hoc* comparisons this observation did not reach statistical significance (2 vs. 3 freeze/thaw cycles *P* = 0.707; 2 vs. 4 freeze/thaw cycles *P* = 0.378 and 2 vs. 5 freeze/thaw cycles *P* = 1.00).

The IL-10 data were non-normally distributed and non-transformable. Using the Friedman test, a difference in IL-10 concentration with number of freeze/thaw cycles was also observed (*P* = 0.005). A Wilcoxon signed rank test comparing 2 and 3 freeze/thaw cycles revealed a difference in the concentration of IL-10 (*P* = 0.005). However, following Bonferroni adjustment required significance levels (*P* = 0.008) were no longer met. Comparisons with 4 or 5 freeze/thaw cycle groups were not different (*P* = 0.105 and *P* = 0.625, respectively).

The TNF-α data were non-normally distributed and non-transformable. A Friedman test showed no difference in TNF-α concentrations with number of freeze/thaw cycles observed (*P* = 0.690).

The median and IQR for molecules relative to the number of freeze/thaw cycles are listed in Table 4.

**Table 4:** Median (interquartile range) concentrations of cytokines measured for each freeze/thaw cycle.

**Freeze/thaw cycle**	**Interleukin 1 receptor antagonist (pg/ml)**	**Interleukin 1β (pg/ml)**	**Interleukin 10 (pg/ml)**	**Tumor necrosis factor α (pg/ml)**	**Interleukin 1 receptor antagonist/interleukin** **1β ratio**
2	3,616 (1,298–9,704)	1,657 (598–4,334)	60.89 (23.64–108.7)	6.270 (0.01–7.765)	1:2.65 (1.72–3.13)
3	2,691 (1,511–5,306)	1,189 (410–2,101)	99.09 (68.19–158.8)	0.815 (0.16–3.320)	1:2.26 (1.33–6.90)
4	2,783 (863–5,491)	2,743 (1,545–11,019)	76.14 (57.69–95.94)	0.66 (0.055–6.418)	1:0.72 (0.38–1.63)
5	2,576 (773.9–4,873)	2,670 (1,543–4,806)	76.41 (50.76–98.86)	1.22 (0.257–4.978)	1:1.10 (0.48–1.0)

For each freeze/thaw cycle, the median and IQR for IL-1β/IL-1Ra ratio were calculated and are listed in Table 4. The IL-1β/IL-1Ra ratio data were non-normally distributed and non-transformable. No correlation between the number of freeze/thaw cycles and IL-1β/IL-1Ra ratio was found.

### Hematology and Blood Biochemistry

Hematological and blood biochemical analyses determined that a systemic inflammatory response was not present in any of the horses. In all horses, total white blood cell count (mean 8.9 ± SD 2.1 × 10^9^/L, normal range 5.5–12.5), fibrinogen (mean 1.5 ± SD 0.2 g/L, normal range 1.0–4.0), serum amyloid A (mean 0.3 ± SD 0.8 mg/L, normal range <7), total protein (mean 66.2 ± SD 5.0, normal range 53–75 g/L) and albumin (mean 33 ± SD 2.2 g/L, normal range 27–39) concentrations were within normal reference ranges. Blood from one horse had a mildly increased globulin concentration (45 g/L, normal range 20–40) and a slightly reduced albumin:globulin (A:G) ratio of 0.7 (mean 1.0 ± SD 0.2, normal range 0.8–1.9). However, with no further evidence suggestive of a systemic inflammatory response this horse's data remained in the analyses.

## Discussion

### Commercial and Non-commercial ACS Preparations: Cytokine Analysis

Our results support the hypothesis that the cytokine profile in a non-commercial ACS product is comparable to that of ACS harvested using a commercial method. A simpler, more cost-effective method of ACS production would allow for greater use of ACS in equine veterinary practice. At our hospital, the wholesale cost of the commercial kit is significantly greater (~$600 AUD) than the cost associated with the alternate method ($40 AUD). The production of IL-1Ra occurred in similar concentrations in both the commercial and non-commercial products. Immunoglobulin binding to monocytes has been shown to activate monocytes and stimulate the production of IL-1Ra alone compared to lipopolysaccharide activation which has been reported to induce both IL-1β and IL-1Ra production; suggesting that monocyte activation is dependent upon external factors (17). The complexity of the method of monocyte activation has led to investigation of the physicochemical induction of IL-1Ra through the interaction of whole blood with CrSO_4_ coated glass beads (8). Exposure of blood to treated glass beads has been described to result in a strong and rapid increase in the synthesis of IL-1Ra and IL-10 (8). However, an increase in cytokine concentrations has also been shown to occur with incubation alone (14, 18–20), suggesting that monocyte activation may be a consequence of thermal induction rather than interactions between whole blood and glass beads. The results of our study are consistent with previous studies which found no difference in the increase of IL-1Ra concentration following incubation of whole blood with and without chromium sulphated glass beads (14, 18–20).

Interleukin 10 is considered to have an important role in the anti-inflammatory effect of ACS products (8), likely by inhibition of IL-1 production and enhancement of IL-1Ra production by monocytes (12). As in this study, no difference in concentrations of IL-10 were found in other studies when blood was incubated for 24 h in the presence or absence of glass beads (14, 18, 20). However, in one study incubation was required for a 2.2-fold increase in IL-10 concentration to occur (8).

Tumor necrosis factor α and IL-1β are pro-inflammatory cytokines which are involved in pathways that have a negative impact on joint health (6). Consequently, increased concentrations of these cytokines in ACS products may lead to a reduced therapeutic benefit. While TNF-α concentration has been shown to increase in a commercial ACS product (18), in our and a previous study (14) a difference in TNF-α concentrations between production systems was not observed. A difference in the concentration of IL-1β with the two production systems was also not noted in previous reports (14, 18). Collectively, the findings of the current and previous studies (14, 18, 20); indicate that cytokine concentrations do not depend on the production method (commercial vs. non-commercial) and it is possible a comparable therapeutic effect may occur with both products. Investigation of the clinical responses to the non-commercial ACS products is required.

Due to the role of IL-1β as an inflammatory mediator of osteoarthritis, the production of this cytokine in an ACS system could influence the potential therapeutic benefit. While IL-1β is effective at low concentrations, higher concentrations of IL-1Ra are required to inhibit its detrimental action (17). Consequently, the IL-1β/IL-1Ra ratio is used to gauge the potential therapeutic efficacy of ACS products and a minimum IL-1β/IL-1Ra ratios of 1:100 for human chondrocytes and 1:30 for human synoviocytes have been proposed to inhibit 50% of IL-1β effects (21). To date no therapeutic IL-1β/IL-1Ra ratio has been determined for horses (11). While there was a positive correlation between IL-1β and IL-1Ra for the commercial containers this was not the case for the non-commercial containers. This positive correlation of IL-1β to IL-1Ra for the commercial method mirrors a similar correlation observed in joints with naturally occurring osteoarthritis, which may be due to a counter induction of IL-1Ra, in response to an increase in IL-1β (22). Despite the presence of positive correlation for the commercial containers, both production systems had increases of IL-1Ra in comparison to IL-1β resulting in IL-1β/IL-1Ra ratios below the 1:100 considered to be of therapeutic value (1:2.65 and 1:1.34, respectively). The results from this study are similar to previous work (19), which found no difference observed between the production method of ACS and IL-1β/IL-1Ra ratios. Given the limited increase in IL-1Ra concentration observed in both products in the current study and a previous study (18), the therapeutic efficacy of ACS should be questioned despite some evidence of therapeutic effects in humans and horses (10, 23, 24). The low concentration of IL-1Ra observed in this study suggest that irrespective of production method, other autologous factors may influence therapeutic outcome. It is possible that beneficial responses to ACS may be due to the concurrent synchronous change of various molecules rather than the increase in one single anti-inflammatory cytokine. Other components that have been identified as constituents of ACS include growth factors such as transforming growth factor beta 1 (TGF-β1), platelet-derived growth factor (PDGF) and Insulin like growth factor-1 (IGF-1) (13, 25). Insulin like growth factor 1 within ACS products has been reported to be associated with a therapeutic benefit of ACS administration (24). An improved effect on the repair processes in IL-1β depleted chondrocytes was seen when IL-1Ra was combined with IGF-1 *in vitro* (26) and in equine joints with experimentally created cartilage defects (27). These findings suggest that the combination of identified and possibly unidentified ACS components are responsible for exerting an anti-inflammatory effect in synovial spaces.

### Commercial and Non-commercial ACS Preparations: Bacteriology

Our hypothesis that the alternate production method would be associated with greater prevalence of positive bacterial cultures is not supported by our data. Of the 10 samples undergoing non-commercial ACS production none provided evidence of microbiological contamination. In contrast, two of the 10 commercial samples had evidence of microbiological contamination. One sample grew a *Micrococcus* sp. and the other grew a *Streptococcus* sp. *Micrococcus* sp. are gram positive cocci, representing prevalent skin isolates in horses (28) and people (29). *Micrococcus* sp. have been isolated before and after aseptic preparation from clipped and non-clipped arthrocentesis sites in horses (28) as well as over the sites of catheter insertion into the jugular vein (30). The genus Micrococcus is not considered to be pathogenic, however infections have been recorded in human patients (29). *Streptococcus* sp. are gram positive cocci bacteria, which are known to be opportunistic pathogens that can cause both local and systemic infections in animals, including horses (31). In a study of disinfection methods for sites of jugular venipuncture in horses, *Streptococcus* sp. were cultured in some horses after disinfection (30). From our experience single colony growths of bacterial isolates from clinical samples are typically representative of contamination and often given less relevance. However, in this instance as ACS is considered an aseptic product, the presence of microbial pathogens should be considered an important finding. The presence of microbiological pathogens in ACS from commercial kits is possibly attributable to the methodology of serum collection, processing or occurred in the laboratory setting. Both the commercial and non-commercial products were collected from the same venipuncture site, making contamination at this time unlikely as only the commercial product had evidence of microbial contamination. During the processing of the commercial product puncture of the proprietary syringe's injection port is required. The injection port is seated below the level of a plastic cover, making disinfection difficult to achieve, which may have led to the observed level of contamination (Figure 1). Disinfection of the injection port could have been achieved using aerosolized alcohol, but this was not pursued. Other possible explanations for the bacterial growth observed could be contamination in the laboratory setting either during aliquoting of the serum into containers or during plating onto culture medium. The use of a laminar flow cabinet during handling of either product may also reduce the risk of microbial contamination. In addition to disinfection protocols of contact sites, handling of production kits and samples in specialized environments should also be considered to avoid contamination.

### Effect of Freeze/Thaw Cycles on Cytokine Concentration

Our results supported the hypothesis that repeated freeze/thaw cycles reduced the concentration of IL-1Ra, however this only occurred at the 5th freeze/thaw cycle. Optimal sample storage conditions that maintain an adequate concentration of cytokines within ACS is not known. Cytokines degrade or are released from cells after sample collection (32). Our results support our hypothesis that increasing freeze/thaw cycles will decrease cytokine concentrations. A negative association been the number of freeze/thaw cycles and the concentration of IL-1Ra was present. This was characterized by a decreasing concentration of IL-1Ra with increasing freeze/thaw cycles, which reached significance at 5 freeze/thaw cycles. Thermal stability of human IL-1Ra has previously been reported to be unaffected by multiple freeze/thaw cycles (33); however, in another study IL-1Ra concentration decreased following 2 freeze/thaw cycles (34). Increased concentrations of IL-1Ra in ACS have been associated with increased therapeutic effect (24), thus decreasing concentrations of IL-1Ra following multiple freeze/thaw cycles may result in decreased therapeutic effect. However, the minimum concentration of IL-1Ra to achieve a beneficial effect is currently unknown. Unlike IL-1Ra, no difference in IL-10 concentration between the number of freeze/thaw cycles was found in this study. In human studies, there is contrasting evidence of the thermal stability of IL-10, with reports of concentration depletion following multiple freeze/thaw cycles (33, 35), whilst other authors found evidence of thermal stability (36). The importance of IL-10 concentration on the therapeutic effect of ACS is unknown (24). However, due to the important anti-inflammatory action of IL-10, it would suggest that thermal stability of this cytokine following repeat freeze/thaw cycles would be therapeutically advantageous.

In this study, IL-1β concentration was associated with the number of freeze/thaw cycles. However, *post hoc* comparisons found no difference between the control (2 freeze/thaw cycles) and 3, 4 and 5 freeze/thaw cycles. This is similar to previous work which found human IL-1β concentration was not affected by up to 6 freeze/thaw cycles (37). Conversely, IL-1β concentrations decreased in samples that underwent 2 freeze/thaw cycles in another study (36). No difference in TNF-α concentration occurred between freeze/thaw cycles in the current study. Tumor necrosis factor α appears to be resilient to freeze/thaw processes; in previous studies, TNF-α concentration was unaffected in up to 6 and 10 freeze/thaw cycles (36, 38). The influence of TNF-α and IL-1β concentration within an ACS product on therapeutic effect has not been quantified in the horse (24). However, due to the known negative effects of TNF-α and IL-1β on joint health (6), a reduction in the concentration of these cytokines would likely be beneficial.

Several scenarios in the clinical setting may result in unintended thawing of samples, such as during shipment of samples, handling errors or due to a malfunctioning freezer. The findings of this study and work by others (32) suggest that if thawing and freezing was to occur up to 3 times it would be unlikely to result in a reduction in the concentration of cytokines of the ACS product.

### Limitations

The large standard-deviation in this data set represents a valid limitation of this study and is likely reflective of individual biological variability in cytokine/molecule responses. In this study the individual variability was adjusted for by setting the horse as a random effect in the multivariable analyses. Despite the reported number of horses being similar to (14) or double that of previous studies (18), the current sample size may not have been sufficient to avoid type 2 errors and larger studies are required to observe an effect of production method. *In vivo* work is also needed to assess the efficacy of the non-commercial product, compared to the commercial product before conclusions of clinical efficacy can be made.

No unfrozen or 1 freeze/thaw data set was included in this study due to the need to re-aliquot samples for transport to the laboratory, resulting in 2 freeze/thaw cycles being used as the control. The finding that cytokine concentrations decreased with increasing freeze/thaw iteration would suggest that cytokine concentrations would have been higher in unfrozen samples or samples after 1 freeze/thaw. However, given that only after three repetitions a significant difference was observed a clinically relevant impact is likely absent and the validity of this conclusions is not affected by the lack of an unfrozen or once frozen sample.

The long-term storage (18 months) of samples prior to analysis may have affected cytokine concentrations. While human cytokines when stored at −80°C remain stable for up to 2 years (39), levels decrease after 8 days when stored at −20°C (36). Unfortunately, no comparative investigations have been performed in horses to establish if the same holds true. ACS samples are typically stored at −20°C for use in veterinary practice due to practical considerations which informed decisions on methodology.

## Conclusion

Our findings suggest that in geldings, the non-commercial method of ACS production results in a comparable cytokine profile than when a commercial preparation kit is used. Further *in vivo* research assessing efficacy of this product should be performed prior to utilization of this method in a clinical setting. When storing ACS samples for future use it is recommended to reduce the number of times a sample undergoes a freeze/thaw cycle prior to *in vivo* use, however 1–3 freeze/thaw cycles is unlikely to affect cytokine concentrations.

## Data Availability Statement

The raw data supporting the conclusions of this article will be made available by the authors, without undue reservation.

## Ethics Statement

The animal study was reviewed and approved by Charles Sturt University Animal Care and Ethics Committee.

## Author Contributions

JH responsible for primary research, including sample collection and manuscript preparation. KH secondary doctorate supervisor and assisted with manuscript preparation. SH performed cytokine analysis on serum samples. RL primary doctorate supervisor and assisted with manuscript preparation. All authors contributed to the article and approved the submitted version.

## Funding

This work was supported by Faculty of Science and Health, Charles Sturt University.

## Conflict of Interest

The authors declare that the research was conducted in the absence of any commercial or financial relationships that could be construed as a potential conflict of interest.

## Publisher's Note

All claims expressed in this article are solely those of the authors and do not necessarily represent those of their affiliated organizations, or those of the publisher, the editors and the reviewers. Any product that may be evaluated in this article, or claim that may be made by its manufacturer, is not guaranteed or endorsed by the publisher.
