# Supplementary material for: Effects of Production Method and Repeated Freeze Thaw Cycles on Cytokine Concentrations and Microbial Contamination in Equine Autologous Conditioned Serum
Source: Front Vet Sci. 2021 Nov 25;8:759828. doi: 10.3389/fvets.2021.759828 (PMC8656450; doi:10.3389/fvets.2021.759828)
Supplement: Supplementary file 1 [file Data_Sheet_1.docx]

Supplementary Item 1 – standard validation curve IL-1RA FMIA
